# Using Photovoice to Understand Barriers to and Facilitators of Cardiovascular Health Among African American Adults and Adolescents, North Carolina, 2011–2012

**DOI:** 10.5888/pcd12.150062

**Published:** 2015-10-01

**Authors:** Sarah Kowitt, Briana Woods-Jaeger, Jesse Lomas, Tamara Taggart, Linden Thayer, Sussie Sutton, Alexandra F. Lightfoot

**Affiliations:** Author Affiliations: Briana Woods-Jaeger, University of Missouri-Kansas City School of Medicine, Kansas City, Missouri; Jesse Lomas, Wake Forest University, Winston-Salem, North Carolina; Tamara Taggart, Linden Thayer, Alexandra F. Lightfoot, University of North Carolina at Chapel Hill, North Carolina; Sussie Sutton, Retired Health Educator, LaGrange, North Carolina.

## Abstract

**Introduction:**

Cardiovascular disease is the leading cause of death in the United States, and mortality rates are higher among African Americans than among people of other races/ethnicities. We aimed to understand how African American adults and adolescents conceptualize cardiovascular health and perceive related barriers and facilitators.

**Methods:**

This qualitative study was conducted as formative research for a larger study, Heart Healthy Lenoir, which aimed to reduce cardiovascular disease disparities among African Americans in eastern North Carolina, part of the widely-known “stroke belt” that runs through the southeastern United States. Using photovoice, a community-based participatory research method, we conducted eight 90-minute photovoice sessions with 6 adults and 9 adolescents in Lenoir County, North Carolina. Topics for each discussion were selected by participants and reflected themes related to cardiovascular health promotion. All sessions were transcribed and coded using a data-driven, inductive approach.

**Results:**

Participants conceptualized cardiovascular health to have mental, spiritual, and social health dimensions. Given these broad domains, participants acknowledged many ecological barriers to cardiovascular health; however, they also emphasized the importance of personal responsibility. Facilitators for cardiovascular health included using social health (eg, family/community relationships) and spiritual health dimensions (eg, understanding one’s body and purpose) to improve health behaviors.

**Conclusion:**

The perspectives of African American adults and adolescents elicited through this formative research provided a strong foundation for Heart Healthy Lenoir’s ongoing engagement of community members in Lenoir County and development and implementation of its intervention to prevent cardiovascular disease.

## Introduction

Cardiovascular disease (CVD) is the leading cause of illness and death in the United States (1). By 2030, 40.5% of the US population is projected to have some form of CVD; for African Americans, rates are expected to be higher (2). Until recently, CVD was the primary outcome for assessing heart health. However, in acknowledgment of the importance of preventing CVD, the American Heart Association (AHA) created a new concept, *cardiovascular health*, which is defined by the presence of “ideal health behaviors” (ie, not smoking, engaging in regular physical activity, eating a healthy diet, and maintaining a healthy body weight) and “ideal health factors” (ie, keeping cholesterol, blood pressure, and fasting blood glucose below recommended levels) (3). Given the growing impact of CVD, the AHA set a national goal to improve cardiovascular health by 20% by 2020, with a subgoal of monitoring and improving the cardiovascular health of underserved populations, including African Americans (3).

Research has implicated many ecological factors in CVD disparities that adversely affect African Americans, including racial residential segregation, socioeconomic inequalities, and unequal concentrations of poverty and wealth (4). However, although it may be clear which risk factors predispose African Americans to CVD, it is unclear how African Americans define cardiovascular health. Moreover, although studies have identified factors that increase communities’ CVD risk, less research has focused on identifying protective factors that may improve cardiovascular health. 

In this qualitative study, we aimed to understand how African American adults and adolescents in Lenoir County, North Carolina, conceptualize cardiovascular health and perceive its related barriers and facilitators. The Social Ecological Framework, which posits that health behaviors are affected by multiple levels of influence (eg, institutional, community, interpersonal, and intrapersonal), was used to frame the study and analyze results (5).

## Methods

**Figure 1:**
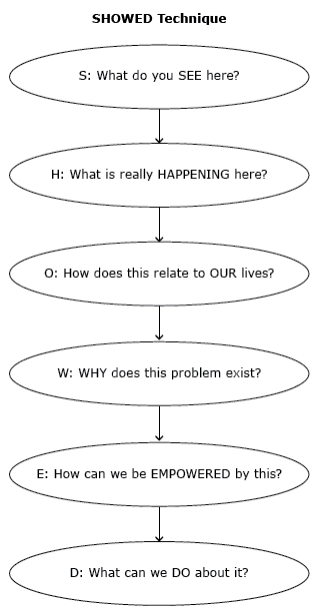
The SHOWED mnemonic.

This study was conducted as formative research for a larger study, Heart Healthy Lenoir (HHL), which aimed to reduce cardiovascular disease disparities among African Americans in eastern North Carolina (6–11). The community-based participatory research (CBPR) methods of photovoice, a process by which people are provided with cameras to take pictures that show community concerns, were used to collect data (12,13). At the heart of photovoice is the SHOWED technique (14) (Figure 1), a dialogue process that draws on Paulo Freire’s notions of empowerment education (15) to bring the perspective and voices of community members into the research process. We chose to use photovoice, because it is an effective tool for engaging communities in identifying public health concerns and potential targets for change as a first step in developing interventions to address them (16).

### Setting and participants

**Figure 2:**
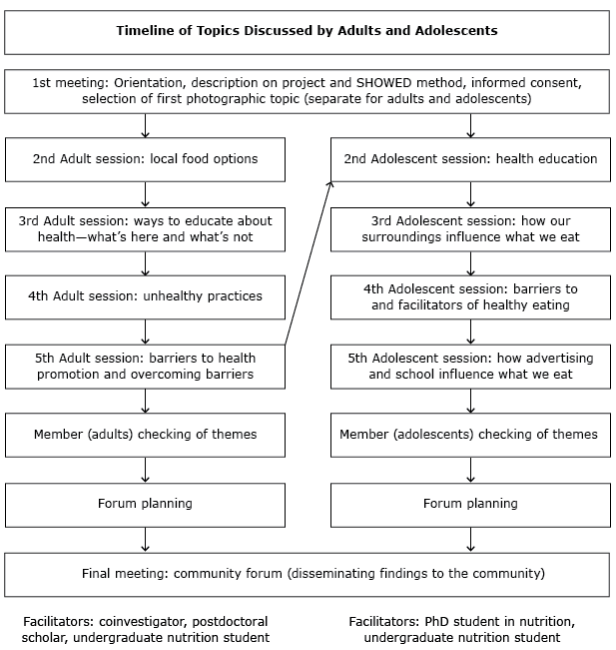
Timeline of topics discussed. The adolescent sessions occurred after the adult sessions (represented by an arrow) and resulted because of adults’ beliefs that any type of community health promotion should include adolescents.

The study was conducted during 2011 and 2012 in Lenoir County, North Carolina, a rural community with a large African American (40%), low-income population (23.2% poverty rate in 2010) that is situated in the widely known “stroke belt” that runs through the southeastern United States (7,17). Institutional review board approval for this research was obtained through the University of North Carolina at Chapel Hill, and written consent from all participants and parents or guardians of participating adolescents was given before participation in discussion groups. We sought participants who were African American, able to commit to attending multiple sessions, and willing to share their perspectives on the health of people in their community. A total of 6 adults (5 female, 1 male) and 9 adolescents aged 14 to 17 (6 female, 3 male) participated in 2 different photovoice projects, each conducted over 6 months with 4 photo discussion sessions each. Participants were recruited through purposive sampling via 2 local community leaders (a community advisory committee member and a participant assisting with HHL formative research). For the adult group, the community leader (a coauthor of this paper) was a former health educator with deep roots in the African American community. Building on her connections in the community, she identified potential adult participants from diverse settings (churches, community-based organizations, rural and urban sections of the county) and invited them to an informational meeting with the research team where a face-to-face recruitment and informed consent process took place (Figure 2). The community leader who recruited the adolescent group was the director of a community-based organization serving African American youth. She identified potential youth participants in her organization and invited them and their parents or guardians to an informational meeting with the research team.

### Data collection

After orientation and providing consent, participants were asked to generate photo assignments related to barriers to and facilitators of heart health. Participants were given a camera and took photos each week of the agreed-upon photo assignment. When the groups reconvened a week later, each participant shared 2 to 3 photographs he or she had taken related to the photo assignment (Figure 2). Each group discussion was moderated by 2 research team facilitators and lasted approximately 90 minutes. Through discussion and voting, the participant group then selected 1 to 2 “trigger” photographs (ie, photographs that could trigger critical reflection) for in-depth discussion. For each trigger photograph, facilitators led the group through a structured discussion using the SHOWED mnemonic to generate new understanding about barriers and strategies for improving cardiovascular health in the community (16). All group discussion sessions were transcribed verbatim and imported into Atlas.ti version 7 (Scientific Software Development GmbH) for further analysis (18).

### Data analysis

Data from the 2 photovoice groups were initially analyzed separately by the photovoice facilitators and member-checked by adolescent and adult participants to ensure the themes that emerged reflected their perspectives. Results from each photovoice project were shared through a joint community forum to present the findings to the community, friends and family, and other stakeholders and to initiate conversations about heart health. Subsequently, these data were reanalyzed jointly in fall 2014. Through a data-driven, inductive approach, the first author read through each transcript several times and developed a set of codes (19), which were systematically applied to all transcripts. After coding, matrices were developed for all topical and interpretive codes to clarify understanding of concepts and relationships between codes. The entire research team discussed emergent themes in team meetings to distill data into key themes. All photographs shared in sessions (n = 126) were analyzed according to photographic-specific codes to identify key themes, such as participants’ descriptions of the photographs, imagery, and connections to the photo assignment. To ensure trustworthiness and rigor, research themes were based on participants’ perspectives and photographs, themes were member-checked with the participants, quotations and photographs were presented to illustrate key themes, a log was kept of all analysis decisions, and repeated sessions allowed us to iterate findings (19).

## Results

Through data analysis of the 8 group sessions, we identified several themes present in both the adolescent and adult groups, organized into 3 interconnected categories: 1) conceptualization of cardiovascular health, 2) barriers to cardiovascular health, and 3) facilitators of cardiovascular health.

### Conceptualization of cardiovascular health

Throughout the group discussions, adult and adolescent participants described cardiovascular health in line with the AHA definition (ie, not smoking, engaging in regular physical activity, eating a healthy diet, maintaining a healthy body weight, and keeping cholesterol, blood pressure, and fasting blood glucose below recommended levels). In addition to these domains, participants also spoke about the importance of mental (ie, depressive and anxiety symptoms, psychological stress, and anger), spiritual (ie, meaning and purpose), and social (ie, belongingness) components of heart health (20,21). For mental health, adults consistently described stress as adversely affecting health behaviors (eg, unhealthy eating, sedentary behavior) and physical health outcomes (eg, hypertension) related to CVD. Stress was described as being so powerful that even if individuals pursued other healthy behaviors (eg, marathon running), they could still end up dying prematurely or unexpectedly. Despite the influence of stress, it was frequently underestimated as a health factor by others in the community. As one adult explained,

I think us as a people, this whole stress thing is new. We just think this is how it is. . . . The whole prejudice thing is so stressful, but we don’t see it as stress. It’s just, we got to do this. My parents did it. . . . But as a people, I don’t think until recently, since it’s everywhere . . . people didn’t look at stress as a health factor. (adult participant)

As the quote illustrates, adult participants described stress as a product of race-based prejudice, as an artifact of everyday life, and as a mindset. Because it was embodied and experienced day-to-day, it was not recognized as a health factor. Only adults discussed stress and its relationship with racism. In explanation of this phenomenon, one adult stated, “The adults lived it [racism]. The children don’t know. They’ve been told about it.” As the quote suggests, adults believed adolescents were not as familiar with encounters of race-based prejudice and any resulting stress.

In addition to mental health, adults also described the roles of faith, meaning, and purpose in their lives (eg, spiritual health) in relation to cardiovascular health. For instance, adults talked about how their faith in God could “take a piece of that cancer,” how Jesus set an example for healthy living, and how understanding one’s larger meaning in life could inspire healthy behaviors. Although adolescents did not explicitly discuss faith and God as connected to cardiovascular health, they did emphasize the importance of family and relationships (ie, social health) and their effect on cardiovascular health behaviors (eg, exercise, diet).

### Barriers to cardiovascular health

Given the broad conceptualization of cardiovascular health dimensions, adult and adolescent participants discussed many barriers to cardiovascular health, which have been organized according to the Social Ecological Framework (5). These barriers included problems with the physical environment, the social environment, a lack of resources, and advertising for unhealthy lifestyles (Table 1).

**Table 1:** Participants’ Use of Photovoice to Demonstrate Barriers to Cardiovascular Health, North Carolina, 2011–2012

Barrier	Description	Photograph
Lack of healthy food in the community	“What I’m showing here is the amount of fast food places on [omitted] Avenue within a mile. I would say less than a half a mile! 4 pizza joints . . . you got Moons, then you got a steak house right next to it. So you get your Chinese food and then your greasy sub sandwiches here. Burger King’s, and I didn’t go up and get the other ones. . . . You have BoJangles, the Mexican joint.” (Adult participant)	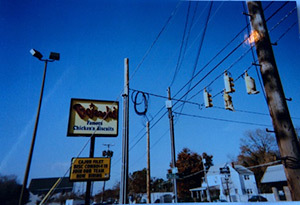
Physical environments can make it difficult to exercise	“We live on a busy road. We live on [omitted] Road, but if you branch off there’s little neighborhoods like this. There’s a branch where you can go ride your bike. But where we live at, you walk out (laughter) you won’t be walkin’ no more.” (Adolescent participant)	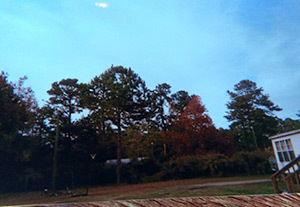
Advertising for unhealthy food	“Have you ever noticed, like when you walk in the store, like most of the time right up front where the cash registers are it’s candy, candy. All the healthy stuff is in the back, and right up front is the candy, so it’s the first thing you see ‘cause everybody knows you’re gonna buy it ‘cause it’s good. It’s advertised good. People like it!” (Adolescent participant)	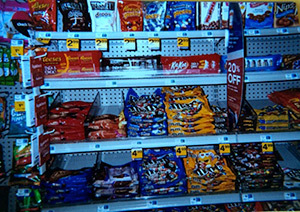
Technology can encourage sedentary behavior	“Yeah, the TV up there it’s like I wanna watch football on that. Yeah, yeah, like no one wants to go outside when you got a big flat screen you can watch anything you want.” (Adolescent participant)	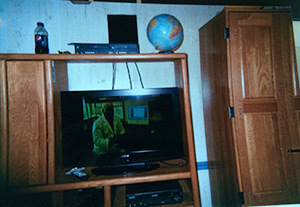
Family members and friends can influence diet and health behaviors	“Basically, my pictures are about pictures of the refrigerator and the cabinet and stuff that’s cooked, because stuff that affects how I eat is what my grandma buys.” (Adolescent participant)	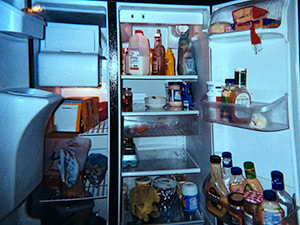

Despite acknowledging these barriers to cardiovascular health, participants attributed responsibility to individuals. Particularly among adults, many participants affirmed that, despite community, institutional, and interpersonal barriers, they were still able to choose healthy behaviors:

But still when you go into a store you still have choices, even based on what’s on sale. Here’s the choice, between sections. What’s on sale here, $2.99 cake or sales at the fruit, produce section. I still have a choice: in the midst of all these regular ones, there was a low-calorie one. (adult participant)

From photographs 1 and 2 in Table 2, the juxtaposition between ecological influences on health decisions and individual responsibility is clear. In the foreground, the convenience, affordability, and accessibility of processed, cheap food is apparent through the sales and advertising for snack foods; in the background, fruits and vegetables are visible, yet they are presented without any type of positive promotion. As many participants emphasized, despite barriers that may make it difficult to be healthy, “it’s up to the person to make the choices.” Accordingly, in talking about instances when adults failed to make healthy choices, participants discussed having a lapse in determination, lacking a “strong mind,” and not staying “conscious” to their true goals. Through these descriptors, the emphasis on mental (ie, psychological willpower and fortitude) and spiritual (ie, purpose, meaning) health is evident.

**Table 2:** Participants’ Use of Photovoice to Illustrate the Tension Between Ecological Influences on Health Decisions and Individual Responsibility, North Carolina, 2011–2012

Photograph Number	Description	Photograph
Photograph 1	Sales of snack foods	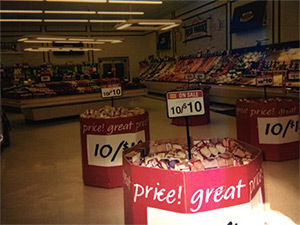
Photograph 2	Sales of cake	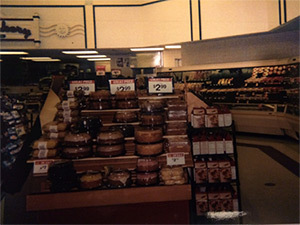

All participants endorsed the idea of choice and individual agency in cardiovascular health behaviors to some extent, yet 2 nuances emerged. First, discussions over individual responsibility for cardiovascular health were at times contentious and one adult participant reported feeling “personally attacked” by her peers for not making enough healthy choices. This sentiment exemplifies the challenges and tensions that participants may feel in having to be responsible for health decisions as well as the judgment and social pressure attached to being healthy. Second, similar to adults, adolescents attributed responsibility for individuals to make healthy choices; however, they described less ability to stay healthy in the future. For instance, although some adolescents reported that they could stay healthy if they got their “priorities straight,” others discussed challenges, such as the inability to choose what foods their parents purchased. For instance, one adolescent reported in reference to the allure of candy and chips, “Because like I’ll try to stay healthy, but there’s always gonna be something that gets you off track.” Thus, although adults described greater agency to be healthy, adolescents explicitly discounted their ability to stay healthy, at least when faced with temptation from unhealthy foods.

### Facilitators to cardiovascular health

Participants also discussed several communitywide strengths and assets that could be used to improve heart health. These facilitators were related to participants’ conceptualization of heart health and included social, spiritual, and mental health dimensions. For instance, given the importance of social health, both adults and adolescents discussed how family and community relationships could be used to promote heart health. Although adults sometimes spoke of making unhealthy choices, they consistently emphasized how their experiences and knowledge could be used to help adolescents make better decisions. As one adult said,

A lifetime full of experiences, good and bad. I have done a lot of bad things in my life that maybe I can prevent somebody else from doin’.

Likewise, adolescents discussed how they often relied on family members and elders to determine what was healthy or not. Although adolescents discussed how adults could sometimes negatively influence their health through poor eating and drinking habits (eg, one adolescent described his grandma as having Pepsi running through her veins), when asked about adolescents’ role models for health, family members were always mentioned. Therefore, both adults and adolescents discussed the importance of family and community members in the behaviors they engaged in and choices they made about their health.

Adults also mentioned that spiritual health dimensions could be used as a means for improving cardiovascular health. Adult participants discussed how understanding one’s purpose and finding meaning in life could improve health behaviors. For example, when asked about how the community could improve adolescent sedentary behavior, one adult said,

When I look at it, it comes to purpose. ’Cause we all have a purpose for doing whatever we do, so each individual have to look within and see and know, ‘Okay, what is my purpose for this?’

Both adults and adolescents mentioned that being in tune with one’s body (ie, becoming aware of physical, mental, spiritual, and social health dimensions) could help them make healthier decisions:

Start from within and what’s there. You know bein’ overweight doesn’t feel good, and your body does, too. (adult participant)

Accordingly, reflection, meditation, and conscious living were all reported as techniques that individuals could use to improve understanding of one’s body, reduce stress, and overcome barriers to cardiovascular health.

## Discussion

Throughout the photovoice discussions, cardiovascular health was conceptualized broadly to include mental, spiritual, and social health dimensions. Participants acknowledged barriers across ecological levels; however, they also emphasized the importance of personal responsibility. Facilitators for cardiovascular health included using social health (eg, family/community relationships) and spiritual health dimensions (eg, understanding one’s purpose and body) to improve health behaviors. There were 3 main takeaway points from the study.

First, in addition to domains included in the AHA definition of cardiovascular health, participants spoke of mental, spiritual, and social health determinants. Within this conceptualization, stress was frequently discussed as an important health factor for cardiovascular health and intertwined with race-based prejudice. Confirming these accounts, several studies emphasized the links between cardiovascular health and stress (22), particularly for African Americans (23). Future research should explore how interventions may best reduce stress, particularly for communities where stress may result from racism and discrimination. Although it may not be possible to eliminate social and environmental stressors that affect cardiovascular health, interventions may incorporate stress management, mindfulness techniques, and coping strategies as mechanisms for reducing their impact.

Second, although participants in this study acknowledged the importance of ecological factors in cardiovascular health, they also emphasized personal responsibility. Public health discourse emphasizes the importance of ecological factors on health conditions; however, as evidenced by this study, this may not translate into individuals’ acceptance of these ideas. Several studies confirm that many Americans endorse individual responsibility over ecological factors (24,25). The reasons behind these beliefs should be clarified in future research, because they may affect individuals’ interactions and engagement with health promotion interventions, particularly those that focus on changing ecological conditions.

Third, participants frequently invoked the importance of social (adults and youths) and spiritual (adults only) health dimensions in improving communitywide health promotion. In particular, relationships between parents and adolescents were emphasized as strategies to improve the community’s cardiovascular health. Several studies highlighted the importance of social and spiritual dimensions in improving cardiovascular health (26–28), but few interventions take a strengths-based approach when targeting health outcomes in the general community (29). Moreover, many interventions are tailored to high-risk groups, such as adults, whereas primary prevention may be more effective beginning in adolescence when health behaviors and coping mechanisms are developed (30). Given the importance of both adults and adolescents in cardiovascular health promotion, a life course perspective, which accounts for adolescence as a critical period in behavioral development, may be helpful when designing and implementing cardiovascular health interventions and policies (eg, population-based early-life intervention programs) and identifying positive developmental health assets for CVD prevention.

Our study has several limitations. First, the small sample size and purposive sampling design may limit generalizability. Second, although adult participants chose to speak more generally about CVD, adolescent participants focused primarily on a specific health behavior — healthy eating. Despite these limitations, the study increases understanding of people’s perspectives on cardiovascular health in the United States. Specifically, across the life course, African American adults and adolescents conceptualized cardiovascular health to include mental, social, and spiritual domains. These domains, in turn, acted as barriers to and facilitators of engaging in health-promoting behaviors. Future research could build on these and other findings from the HHL study (6–11) to explore not only how best to target and reduce CVD disparities but also how to improve communitywide cardiovascular health.
